# Cuproptosis-related lncRNAs predict the clinical outcome and immune characteristics of hepatocellular carcinoma

**DOI:** 10.3389/fgene.2022.972212

**Published:** 2022-09-23

**Authors:** Hongfei Zhu, Feifei Mao, Kang Wang, Jinkai Feng, Shuqun Cheng

**Affiliations:** ^1^ Tongji University Cancer Center, Shanghai 10th People’s Hospital, School of Medicine, Tongji University, Shanghai, China; ^2^ Department of Hepatic Surgery VI, Eastern Hepatobiliary Surgery Hospital, Second Military Medical University, Shanghai, China

**Keywords:** hepatocellular carcinoma, cuproptosis, prognostic signature, tumor microenvironment, immunotherapy

## Abstract

Cuproptosis, as a novel copper-dependent and non-apoptotic form of cell death, is induced by aggregation of lipoylated mitochondrial proteins and the instability of Fe-S cluster proteins. However, the role of cuproptosis-related long noncoding RNAs (CRLncRNAs) in hepatocellular carcinoma (HCC) has not been clearly elucidated. In this study, we identified and characterized cuproptosis-related lncRNAs in HCC. 343 HCC cases from The Cancer Genome Atlas (TCGA) with gene transcriptome data and clinical data were obtained for analysis after the screening. Univariate and multivariate Cox proportional hazards analyses were performed to establish a prognostic cuproptosis-related lncRNA signature (CRlncSig). We established a prognosis-related model consisting of nine cuproptosis-related lncRNAs: GSEC, AL158166.1, AC005479.2, AL365361.1, AC026412.3, AL031985.3, LINC00426, AC009974.2, AC245060.7, which was validated in the internal cohort. High-risk group stratified by the CRlncSig was significantly related to poor prognosis (*p* < 0.001). The area under the receiver operating characteristic curve (AUC) of 1 year, 3 years, and 5 years of survival were 0.813, 0.789, and 0.752, respectively. Furthermore, a prognostic nomogram including CRlncSig with clinicopathologic factors was built with favorable predictive power. In addition, GO and KEGG enrichment analysis suggested that CRlncSig was involved in many carcinogenesis and immune-related pathways. Additionally, we found that tumor microenvironment, immune infiltration, immune function, and drug response were significantly different between the high-risk and low-risk groups based on the risk model. These results highlight the value of cuproptosis-related lncRNAs on prognosis for HCC patients and provide insight into molecular and immune features underlying cuproptosis-related lncRNAs, which might play an important role in patient management and immunotherapy.

## Introduction

Hepatocellular carcinoma (HCC) is the third leading cause of cancer-related death and ranks sixth among all cancers (Forner et al., 2018). Curative therapeutic approaches including liver transplantation, resection, or ablation could only be applied to patients with early-stage disease, while most patients fail to meet the criteria and have a poor prognosis (Lau et al., 2001). The mortality of HCC roughly matches its incidence because of its aggressive nature and limited treatment options (Sung et al., 2021). Thus, uncovering novel therapeutic targets and prognostic factors is an urgent need to improve treatment efficiency and prognosis.

Copper is a basic trace element for human beings, which is involved in various biological processes such as mitochondrial respiration, oxidative stress, and cytotoxicity (Ruiz et al., 2021; Ge et al., 2022). As to cancer, several studies have reported that the Cu concentration in cancer is much higher than that in normal tissues (Blockhuys et al., 2017; Ge et al., 2022). The dysregulation of copper homeostasis has been related to proliferation, angiogenesis, and metastasis, which indicates copper might play a role in tumorigenesis and tumor progression (Babak and Ahn, 2021; Shanbhag et al., 2021; Oliveri, 2022). Moreover, it also had been reported that copper might play a part in immunity and affect the expression levels of programmed death-ligand 1 (PD-L1) (Jones, 1984; Voli et al., 2020). Recently, Tsvetkov et al. found a novel form of cell death termed cuproptosis. The study revealed that increment of copper in cells could induce the aggregation of lipoylated dihydrolipoamide S-acetyltransferase (DLAT) and then affect mitochondrial tricarboxylic acid (TCA) cycle, which finally leads to proteotoxic stress and cell death (Tsvetkov et al., 2022). Metabolic reprogramming of the tricarboxylic acid (TCA) cycle usually comes with the progression of HCC, promoting tumor survival and proliferation in the context of nutrient deprivation and hypoxia (Todisco et al., 2019). So cuproptosis-related genes might be involved in tumor development and progression.

Long noncoding RNAs (lncRNAs) are a type of transcripts longer than 200 nucleotides lacking protein-coding capacity (Clark et al., 2012). And they are closely related to the development of oncogenesis, progression, metastasis, and prognosis in various tumors (Bhan et al., 2017; Wong et al., 2018). However, there are few studies on cuproptosis-related lncRNAs (CRLncRNAs) in HCC patients.

The present study identified cuproptosis-related lncRNAs and constructed a prognostic signature from these lncRNAs, which was associated with mutation landscape, the tumor microenvironment, and immunotherapy response of HCC patients. Gene enrichment analysis was also carried out to explore potential mechanisms.

## Materials and methods

### Data collection and processing

First, RNA-sequence data (50 normal samples and 374 tumoral samples), gene mutation data (n = 364), and clinical data (n = 377) of HCC patients were derived from the TCGA database (https://portal.gdc.cancer.gov/). The transcripts/genes expression abundance are estimated by STAR and RSEM. After eliminating the normal samples, 19895 mRNA and 16773 lncRNAs were identified in LIHC data using annotation of GENCODE project (v22) (Frankish et al., 2019). We then screened 19 cuproptosis-related genes from previous literature (Supplementary Table S1), and expression data were obtained for these genes in TCGA LIHC (Supplementary Table S2). 977 CRLncRNAs whose expression was correlated to cuproptosis-related genes were identified by Pearson correlation analysis (|R2 | > 0.4, *p* < 0.001). Clinicopathological factors, including age, gender, TNM stage, pathologic grade and complete survival information were also extracted. Disease-free survival (DFS) was obtained from the previous study (Liu et al., 2018). Samples with survival time < 30 days were excluded. Finally, 343 cases with gene transcriptome data and clinical data were obtained for analysis.

### Development of the cuproptosis-related lncRNAs signature

A total of 343 samples with the survival data and expression data were randomly allocated to the training sets (n = 241) and validation set (n = 102) in a 7:3 ratio. Univariate Cox regression analysis was performed to screen CRLncRNAs associated with prognosis in the training set. Then these lncRNAs were analyzed by the least absolute shrinkage and selection operator (LASSO) algorithm with 1000 cycles for the best subset of prognostic lncRNAs, and a cuproptosis-related lncRNAs signature (CRlncSig) was constructed. Risk score = ∑ (coef (β)*EXP_β_), where β represents each selected lncRNA. Patients were assigned to high-risk and low-risk groups with the median risk score as the cutoff value. Kaplan-Meier survival analysis was performed to validate the clinical relevance between the two groups. The ROC curve and c-index were used to assess the predictive power of the model. Stratified analysis was conducted to further assess the additional prognostic value of CRlncSig.

### Validation of the CRlncSig

Baseline characteristics were checked between training sets and validation set. The patients in the validation set were grouped with the same method in the training set and validated using Kaplan-Meier survival analysis and risk plot.

### The independently prognostic value of CRlncSig

Univariate and multivariate Cox regression analyses were used to confirm predictive power. Additionally, the correlation between CRlncSig and clinical characteristics was explored by chi-square test using TCGA.

### Construction of nomogram

Risk score combined with the clinicopathological factor of age, gender, grade, and stage were used to construct a nomogram to predict the 1-, 3-, and 5-year survival of HCC patients. The calibration curve was used to test agreement between the actual overall survival (OS) and those predicted by the nomogram.

### Functional enrichment analysis of risk score-associated genes

Gene Ontology (GO) enrichment and Kyoto Encyclopedia of Genes and Genomes (KEGG) pathway analysis were performed to identify significant module using the “clusterprofiler” R package with adjusted *p* value <0.05.

### Prognostic analysis of the tumor mutational burden

Somatic mutations were analyzed by “maftools” R package and illustrated in waterfall plots. TMB of each sample was calculated according to the definition of the total number of variations per million bases via Perl script (version: 5.30.2) (https://www.perl.org/). According to the median value of the TMB, patients were divided into the high-TMB group and the low-TMB group. Then we merged the mutation data with survival information and performed the Kaplan-Meier survival analysis for the two groups.

### Immune-related analysis of CRlncSig

We used the single-sample gene set enrichment analysis (ssGSEA) algorithm via R packages (limma, GSVA and GSEABase) to assess immune function between high- and low-risk groups based on CRlncSig (Hänzelmann et al., 2013). ESTIMATE and CIBERSORT algorithm was performed to assess the proportions of components in the tumor microenvironment (TME) and immune cell infiltration (Yoshihara et al., 2013; Newman et al., 2015). Then we explored the relationship between the expression level of immune checkpoint genes and the two groups. Immunophenoscore (IPS) was further obtained from The Cancer Immunome Atlas (https://tcia.at/home) and used to assess the clinical response to immunotherapy between the two groups (Charoentong et al., 2017).

### Significance of the CRlncSig in drug sensitivity

Fifty percent maximum inhibitory concentration (IC50) values of different groups for various antitumor drugs recommended for hepatocellular carcinoma were calculated via “pRRophetic” and “ggplot2” R package. The IC50 was then compared between low- and high-risk groups by Wilcoxon signed-rank test.

### Statistical analysis

R version 4.0.2 was used to analyze the data and visualize the results. Clinicopathological parameters were compared using t-tests and chi-square tests. Spearman or Pearson correlation coefficients were performed to evaluate relationships between variables. Survival curves were created by the Kaplan-Meier method and compared by log-rank test. Univariable and multivariable analyses were performed using Cox regression models to determine prognostic factors for DFS and OS. Statistical significance was set at *p* < 0.05.

## Results

### Construction of the CRLncRNAs predictive signature

**FIGURE 1:**
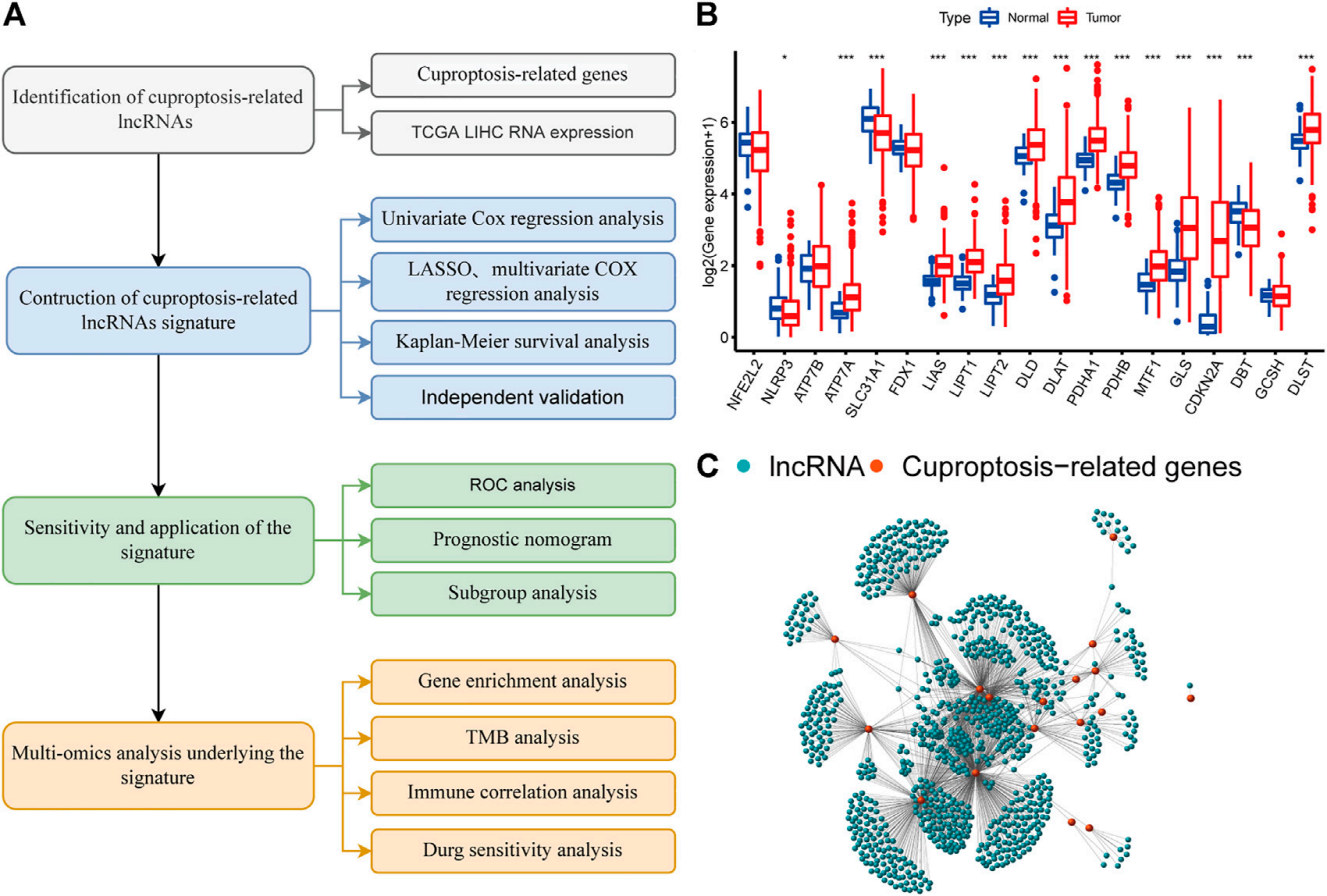
A screen of the differentially expressed cuproptosis-associated lncRNAs in hepatocellular carcinoma (HCC) **(A)** Flowchart of the present research. **(B)** Differential expression of cuproptosis-related genes in normal and HCC tissue **(C)** Network graph of cuproptosis-associated lncRNAs.

The flow chart of this study is shown in Figure 1A. We curated a catalog of 19 cuproptosis-related genes from previous reports (Supplementary Table S1) (Huang et al., 2015; Deigendesch et al., 2018; Tsvetkov et al., 2022). Functional annotations are shown in Supplementary Table S1. Fifteen of these genes showed significant differences (*p* < 0.05) between tumor and normal tissues in LIHC patients from TCGA (Figure 1B). The correlation between cuproptosis-related genes and prognosis of HCC patients is shown in Supplementary Figure S1.

**FIGURE 2:**
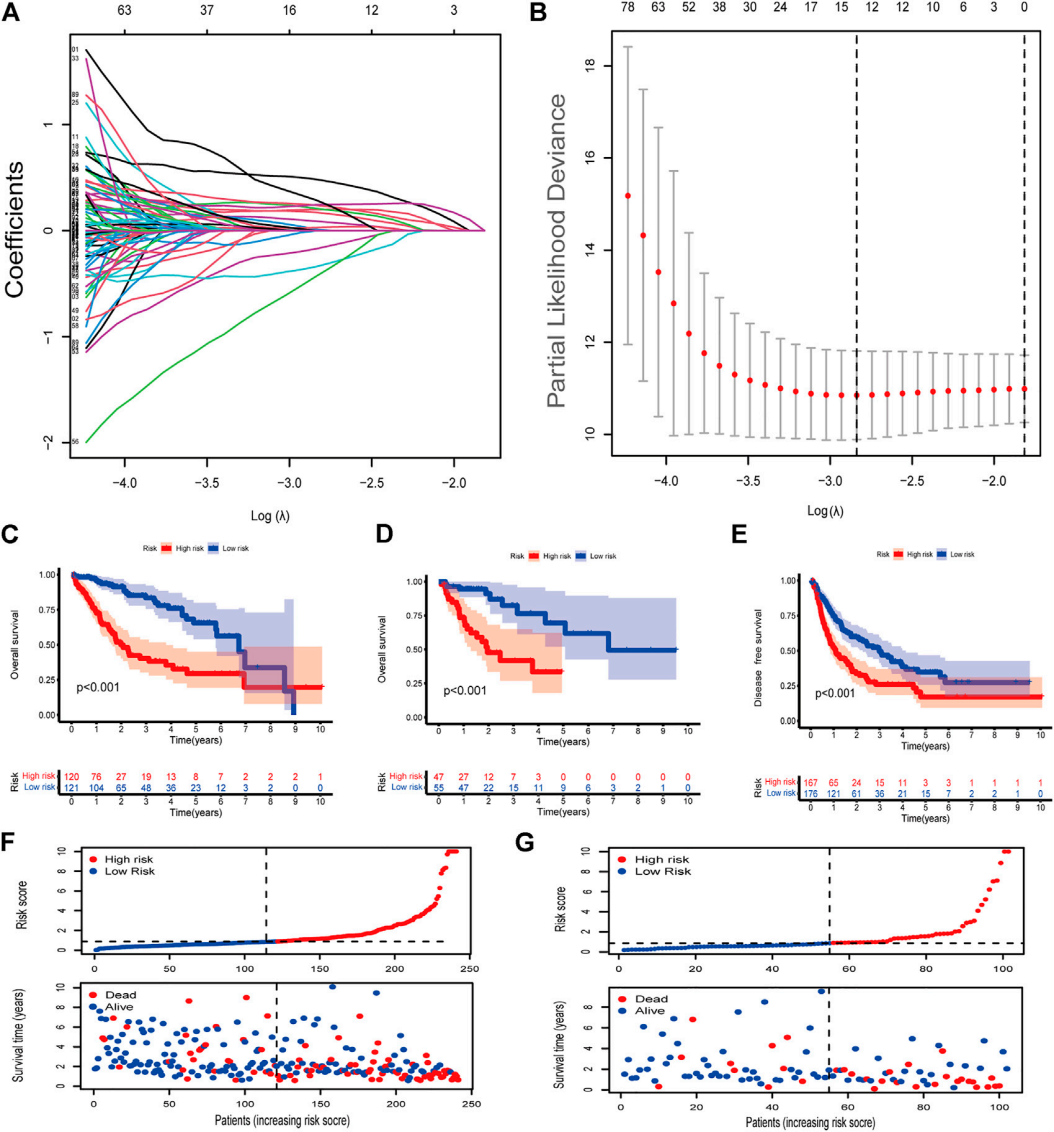
Identification of cuproptosis-associated lncRNAs with prognostic value in hepatocellular carcinoma (HCC) patients **(A,B)** LASSO Cox regression with a 10-fold cross-validation for the prognostic value of the cuproptosis-associated lncRNAs. **(C)** Kaplan-Meier analysis of the OS rate of training set patients in the high- and low-risk groups **(D)** Kaplan-Meier analysis of the OS rate of validation set patients in the high- and low-risk groups. **(E)** Kaplan-Meier analysis of the DFS rate of HCC patients in the high- and low-risk groups **(F)** Risk score distribution, survival status for patients in high- and low-risk groups from training set. **(G)** Risk score distribution, survival status for patients in high- and low-risk groups from validation set.

We identified 977 CRLncRNAs (Figure 1C, Supplementary Table S2). Supplementary Table S3 showed the correlation result between lncRNAS and cuproptosis genes. Then 211 CRLncRNAs were found as prognostic factors using univariate COX analysis (Supplementary Table S4). Subsequently, we performed LASSO Cox regression intending to reduce the risk of over-fitting and 13 robust genes were obtained (Figures 2A,B). Multivariate Cox regression was applied to analyze the thirteen genes and nine of them (GSEC, AL158166.1, AC005479.2, AL365361.1, AC026412.3, AL031985.3, LINC00426, AC009974.2, AC245060.7) were then used to construct a prognostic signature for HCC. Supplementary Figure S2 showed the correlation between cuproptosis-related genes and their associated lncRNAs.

### Correlation between CRlncSig and prognosis of HCC patients

The coefficients of the nine CRlncSig were used to assess the scores for each patient. The risk score was calculated as follows: Risk score = (0.319,888 × expression value of GSEC) + (0.332,438 × expression value of AL158166.1) + (0.40166 × expression value of AC005479.2) + (-0.59091 × expression value of AL365361.1) + (0.764,221 × expression value of AC026412.3) + (0.457,035 × expression value of AL031985.3) + (-0.95334 × expression value of LINC00426) + (-1.61518 × expression value of AC009974.2) + (0.958,349 × expression value of AC245060.7). Then patients were assigned to low- and high-risk groups according to the median value of the risk score. Seventy percent of the 343 patients were randomly divided into the training group and the rest were in the validation group. No significant differences were found in clinical characteristics between the low- and high-groups (Supplementary Table S5). In the training set, patients in high-risk group had shorter overall survival than patients in low-risk group (*p* < 0.001, Figure 2C). This was also validated in the validation set (*p* < 0.001, Figure 2D). Next, we checked the predictive performance in disease-free survival using the dataset with DFS information (Liu et al., 2018). K-M analysis indicated significantly reduced DFS in high-risk patients (*p* < 0.001, Figure 2E). As shown in risk survival status plot, the survival of patients was inversely proportional to the risk score both in training and validation set (Figures 2F,G).

### Evaluation of CRlncSig

**FIGURE 3:**
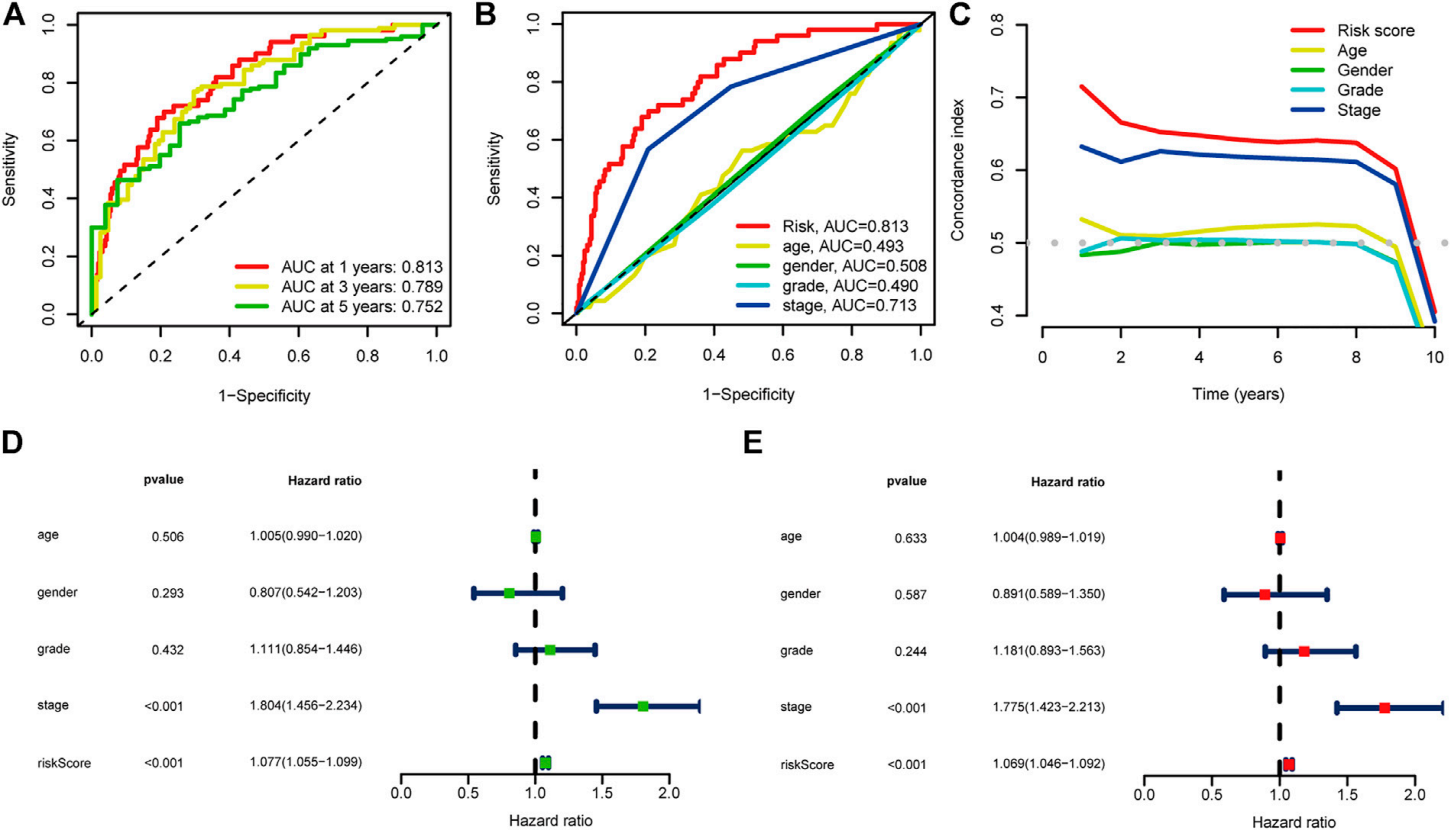
Evaluation of the prognostic cuproptosis-associated lncRNAs signature **(A)** ROC curve and AUCs at 1-year, 3-years and 5-years survival for the predictive signature. **(B)** The ROC curve of the risk score and clinicopathological variables **(C)** C index of the risk score and clinicopathological variables. **(D)** Forest plot for univariate Cox regression analysis. **(E)** Forest plot for multivariate Cox regression analysis.

The time-dependent ROC curve was used to assess the performance of the signature. The area under the ROC curve (AUC) of 1 year, 3 years, and 5 years of survival were 0.813, 0.789, and 0.752, respectively (Figure 3A). The AUC of 1-year survival rate suggested that risk score (0.813) and stage (0.713) possessed a favorable prediction power (Figure 3B). The C-index of the risk score was superior to clinicopathological factors as shown in Figure 3C. The prognostic value of the risk score and other factors were evaluated with univariate and multivariate Cox regression analyses. The risk score and stage were identified as significant independent prognostic factors in both univariate Cox regression analyses (HR = 1.077, 95% CI = 1.055–1.099, *p* < 0.001 and HR = 1.804, 95% CI = 1.456-2.234, *p* < 0.001) and multivariate Cox regression analyses (HR = 1.069, 95% CI = 1.046-1.092, *p* < 0.001 and HR = 1.775, 95% CI = 1.423-2.213, *p* < 0.001) (Figures 3D,E).

### Construction of nomogram

**FIGURE 4:**
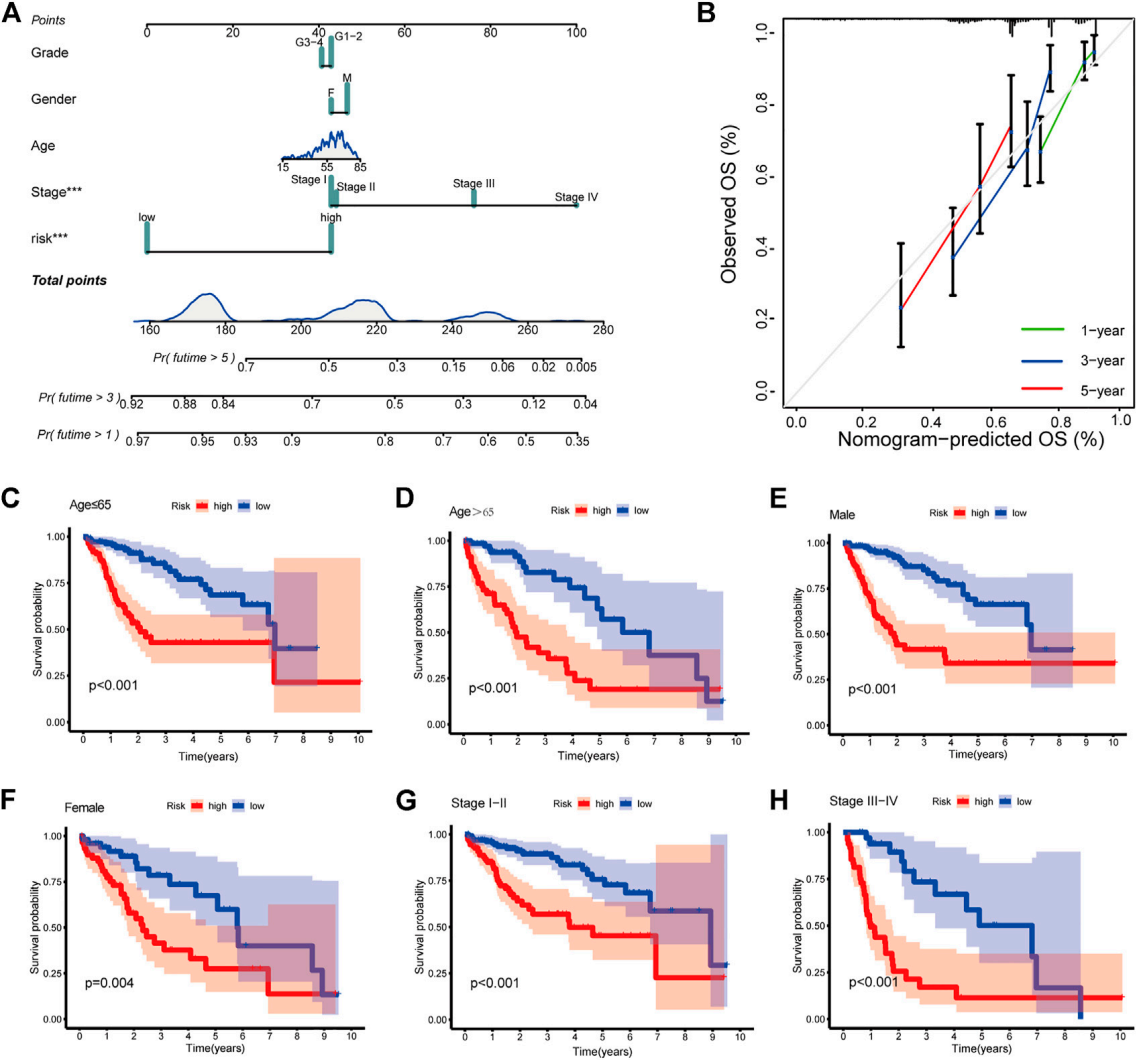
Clinical prognostic nomogram for survival prediction and subgroup analysis **(A)** A nomogram combining clinicopathological variables and risk score predicts 1, 3, and 5 years OS of HCC patients. **(B)** Calibration plots for 1-, 3-, and 5-years survival predictions **(C–H)** Subgroup survival analysis in the high- and low-risk groups, **(C)** Age ≤65 **(D)** Age > 65 **(E)** Male patients **(F)** Female patients **(G)** Stage Ⅰ-Ⅱ **(H)** Stage Ⅲ-Ⅳ.

To provide a quantitative tool for clinical application, we established a nomogram with age, gender, pathological grade, stage, and risk score to predict the overall survival of patients (Figure 4A). The calibration plot showed good consistency between the actual versus predicted rates of the 1, 3, and 5-year OS (Figure 4B).

### Subgroup analysis of clinicopathological variables

At last, to explore the applicability of CRlncSig, patients were assigned into groups according to age, gender, and stage. For each subgroup, patients with high-risk scores had a poor prognosis, which indicated that CRlncSig had good predictive power for all patients (Figures 4C–H).

### Functional and pathway analysis

**FIGURE 5:**
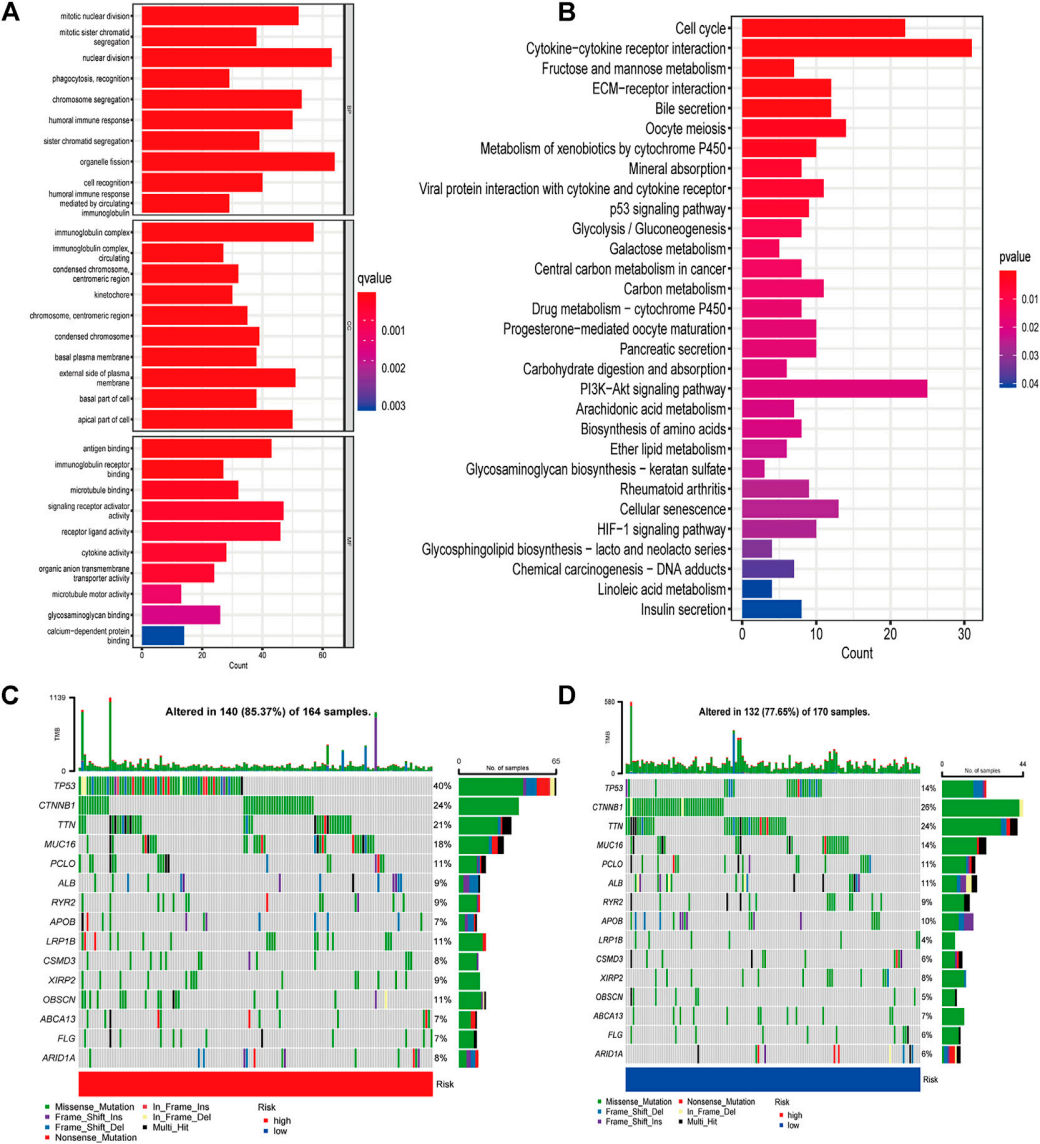
Gene enrichment and TMB in high- and low-risk groups **(A)** GO enrichment analysis **(B)** KEGG enrichment analysis **(C)** Waterfall plots displaying the mutation landscapes of the low-risk group. **(D)** Waterfall plots displaying the mutation landscapes of the high-risk group. TMB, tumor mutation burden; GO, Gene Ontology; KEGG, Kyoto Encyclopedia of Genes and Genomes; BP, Biological process; CC, Cellular component; MF Molecular function.

GO and KEGG analyses were performed to explore the underlying mechanisms of different prognoses between high- and low-risk groups. 1090 differentially expressed genes (DEGs) were obtained between two groups, including 947 upregulated genes and 143 downregulated genes (Supplementary Table S6). The cellular component (CC) of GO enrichment analysis indicated that DEGs were mainly enriched in “immunoglobulin complex”, and “immunoglobulin complex circulating”. Biological process (BP) showed DEGs were mainly associated with “nuclear division”, “phagocytosis, recognition”, and “humoral immune response”. While molecular function (MF) indicated DEGs were mainly concentrated in “antigen binding”, and “immunoglobulin receptor binding” (Figure 5A). According to KEGG pathway analysis, DEGs were found mainly connected with tumorigenesis and cancer progression, such as “ECM-receptor interaction”, “p53 signaling pathway”, “Central carbon metabolism in cancer”, as well as immune-related pathways, such as “HIF-1 signaling pathway” “Cytokine-cytokine receptor interaction”, (Figure 5B). These results suggested that DEGs are involved in both carcinogenesis and immune-related pathways.

### Risk score-associated mutation landscape

Various basic features of somatic mutation data for low- and high-risk groups were shown in waterfall plot (Figures 5C,D). The top three mutated genes were TP53 (40%), CTNNB1 (24%) and TTN (21%) in the high-risk group, while CTNNB1 (26%), TTN (24%) and TP53 (14%) were the most common mutation genes in low-risk group. Missense mutation was the main variant classification in both groups. Then patients were divided into low- and high-TMB groups according to the median value of TMB and a significant survival difference was found between the two groups (Supplementary Figure S3A). The risk score also showed good predictive power when patients were stratified by TMB (Supplementary Figure S3B).

### Immunity analysis of the risk score

**FIGURE 6:**
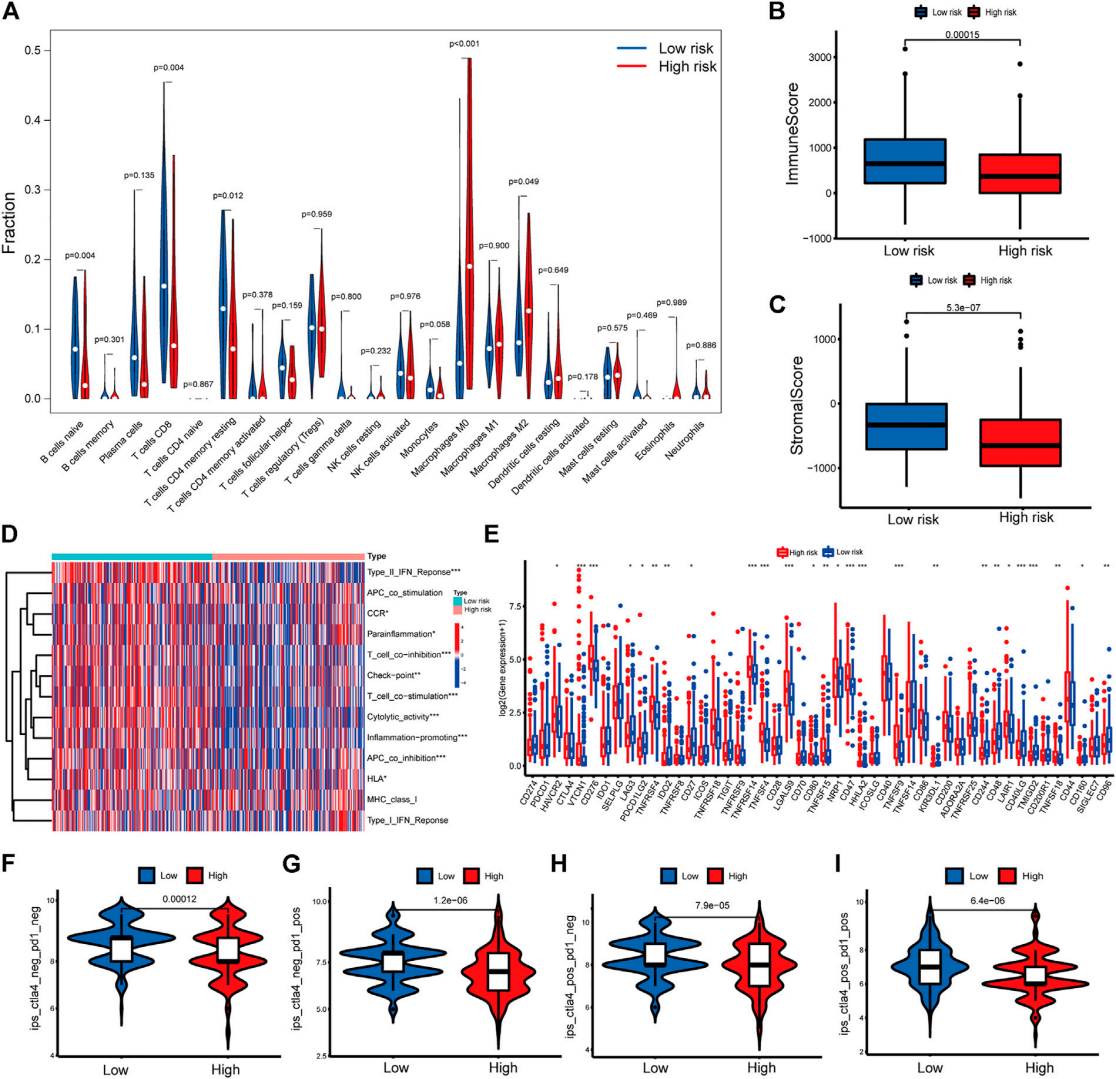
Immune related analysis in high- and low-risk groups **(A)** Differences in the infiltration of immune cells between the high- and low-risk groups. **(B–C)** Comparison of immune score **(B)**, and stromal score **(C)** between the high- and low-risk groups **(D)** The correlation between the signature and 13 immune-related functions. **(E)** Differential expression of immune checkpoint genes between the high- and low-risk groups **(F–I)** IPS values of patients categorized according to risk score of four subtypes [IPS-CTLA4-neg-PD1-neg **(F)**, IPS-CTLA4-neg-PD1-pos **(G)**, IPS-CTLA4-pos-PD1-neg **(H)**, IPS-CTLA4-pos-PD1-pos **(I)**]. IPS, Immunophenoscore. *p < 0.05, **p < 0.01, ***p < 0.001.

To further explore the correlations between risk score and tumor immune cell infiltration, the proportions of 22 immune cell types were compared between the low- and high-risk groups with CIBERSORT algorithm. The results showed that naïve B cells, CD8^+^ T cells (known as main immune effector cells), resting memory CD4^+^ T cells had higher fractions in low-risk group (all *p* < 0.05) while M0 macrophages, M2 macrophages, which were known to exert immunosuppressive functions, had higher fractions in high-risk group (both *p* < 0.05) (Figure 6A). The ESTIMATE algorithms suggested a higher proportion of immune and stromal cells in the low-risk group (Figures 6B,C). Then the immune function was inferred by ssGSEA algorithm. As shown in Figure 6D, Type II IFN (IFN-γ) response, chemokine receptor (CCR), para-inflammation, T cell co-inhibition, checkpoint, T cell co-stimulation, cytolytic activity, inflammation-promoting, antigen-presenting cell (APC) coinhibition and human leukocyte antigen (HLA) were significant difference between two groups, which indicated that immune function was more active in the low-risk group. These results suggested that the signature was not only a predictive marker but also associated with immune function. Next, we explored whether levels of immune checkpoint genes were associated with risk scores. High-risk patients tended to express higher levels of 16 immune checkpoint genes, including HAVCR2, VTCN1, CD276, TNFRSF4, CD27, TNFRSF14, TNFSF4, LGALS9, CD80, TNFRSF15, CD47, HHLA2, TNFSF9, LAIR1, TNFRSF18, CD44, while low-risk patients tended to express higher levels of 10 immune checkpoint genes, including LAG3, PDCD1LG2, IDO2, KIR3DL1, CD244, CD48, CD40LG, TMIGD2, CD160, CD96 (Figure 6E). To access the power of the signature for predicting the response to immunotherapy, immunophenoscore (IPS) calculated and patients in low-risk group had a higher IPS, suggesting that patients in this group might have a better response to immunotherapy (Figures 6F–I).

### Drug response features underlying the CRLncRNAs

In addition to immunotherapy, we also explored the association between the risk score and the efficacy of targeted therapy and chemotherapy for HCC patients. The results suggested that the IC50 of trametinib, talazoparib was positively correlated with risk score and the IC50 of 5-fluorouracil, doxorubicin, gemcitabine, mitomycin C, paclitaxel, sorafenib, sunitinib, tipifarnib, tivozanib, vinorelbine was negatively correlated with risk score (Supplementary Figure S4), which helps explore individualized treatment strategy HCC patients.

## Discussion

HCC is the third leading cause of cancer-related death worldwide. The high molecular and clinical heterogeneity of HCC results in inefficient treatments and poor prognosis (Wörns and Galle, 2014). Integrating multiple biomarkers and clinical features into a single model could improve the accuracy of prediction and help formulate individualized treatment plans when compared with a single biomarker. In the present study, we identified CRLncRNAs and constructed a prognostic signature, which was associated with mutation landscape, the tumor microenvironment, and immunotherapy response of HCC patients. We also explore potential mechanisms through gene enrichment analysis.

We identified 211 CRLncRNAs associated with the overall survival of HCC patients via univariate regressions analysis. Then nine lncRNAs were conformed and developed lncRNA signature for prognostic prediction. Different kinds of predictive lncRNA signatures for HCC patients have been reported in previous studies (Huang et al., 2021; Li et al., 2021; Wang et al., 2021; Yang et al., 2021). Li et al. reported an eight m6A-related lncRNA signature with AUC of 0.633, 0.651, and 0.638 at 1-, 3–5-year (Li et al., 2021). While the highest AUC of the immune- and ferroptosis-related lncRNA signature in 5 years was 0.761 in the study by Huang (Huang et al., 2021). In our study, the lowest AUC in 5 years is 0.753, which indicates this CRLncRNAs signature has strong predictive power.

The critical contribution of this study is to demonstrate the relationship between CRLncRNAs signature and tumor microenvironment. Notably, it is worth noting that TME not only plays a vital role in the development of tumors but also has an important impact on immunotherapy response and overall survival (Hinshaw and Shevde, 2019; Fane and Weeraratna, 2020; Petitprez et al., 2020). Functional enrichment analysis showed that CRLncRNAs were mainly related to cytokine-cytokine receptor interaction, the phosphatidylinositol 3-kinases/protein kinase B (PI3K-AKT) signaling pathway and immune pathways. Cytokines are major regulators of the innate and adaptive immune systems that allow cells of the immune systems to communicate over short distances in paracrine and autocrinefashion (Waldmann, 2018). Cytokine and cytokine receptor interaction networks were regarded as crucial effects on inflammation and oncogenesis (Dranoff, 2004). Cytokines and its receptors, such as tumor necrosis factor and interleukin 6, were important factors in the development of HCC and affected the immunotherapy effect (Kern et al., 2018; Derynck et al., 2021). PI3K-AKT signaling pathway was a classical intracellular signaling receptor to react extracellular stimulators. The PI3K/AKT pathway was dysregulated in both initiation and progression of HCC (Whittaker et al., 2010). To explore whether the signature could predict the efficiency of immunotherapy for HCC patients, we first checked the expression levels of 48 immune checkpoints genes and found that more than half of these genes were related to the risk score. Tumor immune microenvironment was also evaluated between two groups. Patients with high-risk scores had lower proportions of CD8^+^ T cells and higher proportions of M0 macrophages and M2 macrophages, which indicated the roles of CRLncRNAs in regulating the tumor microenvironment. As we know, CD8^+^ T cells are the main effectors in antitumor immunotherapy while M2 macrophages, working as immunosuppressive cells, promote tumor growth and invasion (Pitt et al., 2016). Patients in high-risk group are more likely to be “cold” tumors characterized by resistance to immune checkpoint therapy. IPS, derived from four major gene categories, could work as a superior predictor for immunotherapy (Charoentong et al., 2017). Then we calculated IPS to predict immunotherapy response, patients in low-risk group had a higher IPS, suggesting that patients in this group might respond better to immunotherapy. This is consistent with result of the tumor immune microenvironment analysis.

Recent studies show that cuproptosis is a promising new target for cancer treatment. Copper ionophores have shown promising applications in overcoming drug resistance of cancer cells and targeting cancer stem cells. This is due to the intrinsic selectivity of copper ionophores in preferential induction of cancer cell clusters compared with normal cells (Oliveri, 2022). Another study by Voli et al. showed that copper supplementation promotes PDL1 expression and intratumor copper levels might enhance immunotherapy response (Voli et al., 2020). Our study and previous studies indicate that copper plays an important role in antitumor treatment and immunotherapy. FDX1 is the key regulators of copper ionophore–induced cell death, which encodes a reductase known to reduce Cu^2+^ to its more toxic form, Cu^1+^, and is adirect target of elesclomol (Tsvetkov et al., 2019; Tsvetkov et al., 2022). Recent pan-cancer analysis revealed that FDX1 could be a novel biomarker in the prognosis and immunotherapy in human tumors, which could provide a basis for drug use in certain tumors (Ma et al., 2022; Zhang et al., 2022).

The current study had several limitations. First, we constructed and validate the prognostic model with a single retrospective data source. Second, some well-known prognostic factors for HCC such as AFP and microvessel invasion were not involved in the nomogram because of incomplete data for these parameters. Thus, a prospective study is needed to verify the predictive value of the signature. In addition, functional biological experiments should be carried out to further validate the results.

In summary, the cuproptosis-related lncRNA signature could effectively predict the prognosis and immunotherapy response of HCC patients. Immune analysis verified the association between the risk score and tumor microenvironment. Thus, our results offer a reasonable explanation for the distinct prognoses of patients and provide a rationale for exploring biomarkers and antitumor treatment strategies.

## Data Availability

Publicly available datasets were analyzed in this study. This data can be found here: The Cancer Genome Atlas (https://portal.gdc.cancer.gov/).
