# Supplementary material for: Cuproptosis-related lncRNAs predict the clinical outcome and immune characteristics of hepatocellular carcinoma
Source: Front Genet. 2022 Sep 23;13:972212. doi: 10.3389/fgene.2022.972212 (PMC9538148; doi:10.3389/fgene.2022.972212)
Supplement: Supplementary file 3 [file Table1.DOCX]

**Table S1** A list of cuproptosis -related genes and functional annotation.

| Gene | Gene summary |
| --- | --- |
| FDX1 | FDX1 gene codes for a tiny iron-sulfur protein that transports electrons from NADPH to mitochondrial cytochrome P450 via ferredoxin reductase, which is implicated in the steroid, vitamin D, and bile acid metabolism (1). FDX1 also encodes a reductase known to reduce Cu^2+^ to its more toxic form, Cu^1+^, and to be a direct target of elesclomol (2). |
| LIPT1 | LIPT1 is an enzyme which could activate TCA cycle-related 2-ketoacid dehydrogenase, and inhibit TCA cycle metabolism when it is deficient (3). |
| DLD* | This gene encodes a member of the class-I pyridine nucleotide-disulfide oxidoreductase family. The encoded protein has been identified as a moonlighting protein based on its ability to perform mechanistically distinct functions. In homodimeric form, the encoded protein functions as a dehydrogenase and is found in several multi-enzyme complexes that regulate energy metabolism. However, as a monomer, this protein can function as a protease. Mutations in this gene have been identified in patients with E3-deficient maple syrup urine disease and lipoamide dehydrogenase deficiency. Alternative splicing results in multiple transcript variants. |
| LIAS* | The protein encoded by this gene belongs to the biotin and lipoic acid synthetases family. Localized in the mitochondrion, this iron-sulfur enzyme catalyzes the final step in the de novo pathway for the biosynthesis of lipoic acid, a potent antioxidant. The deficient expression of this enzyme has been linked to conditions such as diabetes, atherosclerosis and neonatal-onset epilepsy. Alternative splicing occurs at this locus, and several transcript variants encoding distinct isoforms have been identified. |
| PDHA1* | The pyruvate dehydrogenase (PDH) complex is a nuclear-encoded mitochondrial multienzyme complex that catalyzes the overall conversion of pyruvate to acetyl-CoA and CO(2), and provides the primary link between glycolysis and the tricarboxylic acid (TCA) cycle. The PDH complex is composed of multiple copies of three enzymatic components: pyruvate dehydrogenase (E1), dihydrolipoamide acetyltransferase (E2) and lipoamide dehydrogenase (E3). The E1 enzyme is a heterotetramer of two alpha and two beta subunits. This gene encodes the E1 alpha 1 subunit containing the E1 active site, and plays a key role in the function of the PDH complex. Mutations in this gene are associated with pyruvate dehydrogenase E1-alpha deficiency and X-linked Leigh syndrome. Alternatively spliced transcript variants encoding different isoforms have been found for this gene. |
| PDHB* | The pyruvate dehydrogenase (PDH) complex is a nuclear-encoded mitochondrial multienzyme complex that catalyzes the overall conversion of pyruvate to acetyl-CoA and carbon dioxide, and provides the primary link between glycolysis and the tricarboxylic acid (TCA) cycle. The PDH complex is composed of multiple copies of three enzymatic components: pyruvate dehydrogenase (E1), dihydrolipoamide acetyltransferase (E2) and lipoamide dehydrogenase (E3). The E1 enzyme is a heterotetramer of two alpha and two beta subunits. This gene encodes the E1 beta subunit. Mutations in this gene are associated with pyruvate dehydrogenase E1-beta deficiency. Alternatively spliced transcript variants have been found for this gene. |
| MTF1 | MTF1 is a metal-binding transcription factor found in eukaryotes. It protects cells from oxidative and hypoxic stress by dual responses to metal excess and deficiency, and its deficiency inhibits epithelial-mesenchymal transition (4). |
| GLS* | This gene encodes the K-type mitochondrial glutaminase. The encoded protein is an phosphate-activated amidohydrolase that catalyzes the hydrolysis of glutamine to glutamate and ammonia. This protein is primarily expressed in the brain and kidney plays an essential role in generating energy for metabolism, synthesizing the brain neurotransmitter glutamate and maintaining acid-base balance in the kidney. Alternate splicing results in multiple transcript variants. GLS is a key enzyme in glutamine metabolism, has been reported to be involved in the occurrence of various cancers (5). |
| CDKN2A* | This gene generates several transcript variants which differ in their first exons. At least three alternatively spliced variants encoding distinct proteins have been reported, two of which encode structurally related isoforms known to function as inhibitors of CDK4 kinase. The remaining transcript includes an alternate first exon located 20 Kb upstream of the remainder of the gene; this transcript contains an alternate open reading frame (ARF) that specifies a protein which is structurally unrelated to the products of the other variants. This ARF product functions as a stabilizer of the tumor suppressor protein p53 as it can interact with, and sequester, the E3 ubiquitin-protein ligase MDM2, a protein responsible for the degradation of p53. In spite of the structural and functional differences, the CDK inhibitor isoforms and the ARF product encoded by this gene, through the regulatory roles of CDK4 and p53 in cell cycle G1 progression, share a common functionality in cell cycle G1 control. This gene is frequently mutated or deleted in a wide variety of tumors, and is known to be an important tumor suppressor gene. Upregulation of CDKN2A in HCC is closely related to poor prognosis (6). |
| NFE2L2* | This gene encodes a transcription factor which is a member of a small family of basic leucine zipper (bZIP) proteins. The encoded transcription factor regulates genes which contain antioxidant response elements (ARE) in their promoters; many of these genes encode proteins involved in response to injury and inflammation which includes the production of free radicals. Multiple transcript variants encoding different isoforms have been characterized for this gene. |
| NLRP3* | This gene encodes a pyrin-like protein containing a pyrin domain, a nucleotide-binding site (NBS) domain, and a leucine-rich repeat (LRR) motif. This protein interacts with the apoptosis-associated speck-like protein PYCARD/ASC, which contains a caspase recruitment domain, and is a member of the NLRP3 inflammasome complex. This complex functions as an upstream activator of NF-kappaB signaling, and it plays a role in the regulation of inflammation, the immune response, and apoptosis. The SARS-CoV 3a protein, a transmembrane pore-forming viroporin, has been shown to activate the NLRP3 inflammasome via the formation of ion channels in macrophages. Mutations in this gene are associated with familial cold autoinflammatory syndrome (FCAS), Muckle-Wells syndrome (MWS), chronic infantile neurological cutaneous and articular (CINCA) syndrome, neonatal-onset multisystem inflammatory disease (NOMID), keratoendotheliitis fugax hereditarian, and deafness, autosomal dominant 34, with or without inflammation. Multiple alternatively spliced transcript variants encoding distinct isoforms have been identified for this gene. Alternative 5' UTR structures are suggested by available data; however, insufficient evidence is available to determine if all of the represented 5' UTR splice patterns are biologically valid |
| ATP7B* | This gene is a member of the P-type cation transport ATPase family and encodes a protein with several membrane-spanning domains, an ATPase consensus sequence, a hinge domain, a phosphorylation site, and at least 2 putative copper-binding sites. This protein is a monomer, and functions as a copper-transporting ATPase which exports copper out of the cells, such as the efflux of hepatic copper into the bile. Alternate transcriptional splice variants, encoding different isoforms with distinct cellular localizations, have been characterized. Mutations in this gene have been associated with Wilson disease which is characterized by copper accumulation. |
| ATP7A* | This gene encodes a transmembrane protein that functions in copper transport across membranes. This protein is localized to the trans Golgi network, where it is predicted to supply copper to copper-dependent enzymes in the secretory pathway. It relocalizes to the plasma membrane under conditions of elevated extracellular copper, and functions in the efflux of copper from cells. Mutations in this gene are associated with Menkes disease, X-linked distal spinal muscular atrophy, and occipital horn syndrome. Alternatively-spliced transcript variants have been observed. |
| SLC31A1* | The protein encoded by this gene is a high-affinity copper transporter found in the cell membrane. The encoded protein functions as a homotrimer to effect the uptake of dietary copper. |
| LIPT2* | This gene encodes a mitochondrial protein that catalyzes the transfer of octanoic acid to lipoate-dependent enzymes such as octanoyl-ACP. Alternative splicing results in multiple transcript variants. |
| DLAT* | This gene encodes component E2 of the multi-enzyme pyruvate dehydrogenase complex (PDC). PDC resides in the inner mitochondrial membrane and catalyzes the conversion of pyruvate to acetyl coenzyme A. The protein product of this gene, dihydrolipoamide acetyltransferase, accepts acetyl groups formed by the oxidative decarboxylation of pyruvate and transfers them to coenzyme A. Dihydrolipoamide acetyltransferase is the antigen for antimitochondrial antibodies. These autoantibodies are present in nearly 95% of patients with the autoimmune liver disease primary biliary cirrhosis (PBC). In PBC, activated T lymphocytes attack and destroy epithelial cells in the bile duct where this protein is abnormally distributed and overexpressed. PBC enventually leads to cirrhosis and liver failure. Mutations in this gene are also a cause of pyruvate dehydrogenase E2 deficiency which causes primary lactic acidosis in infancy and early childhood. |
| DBT* | The branched-chain alpha-keto acid dehydrogenase complex (BCKD) is an inner-mitochondrial enzyme complex involved in the breakdown of the branched-chain amino acids isoleucine, leucine, and valine. The BCKD complex is thought to be composed of a core of 24 transacylase (E2) subunits, and associated decarboxylase (E1), dehydrogenase (E3), and regulatory subunits. This gene encodes the transacylase (E2) subunit. Mutations in this gene result in maple syrup urine disease, type 2. Alternatively spliced transcript variants have been described, but their biological validity has not been determined. |
| GCSH* | Degradation of glycine is brought about by the glycine cleavage system, which is composed of four mitochondrial protein components: P protein (a pyridoxal phosphate-dependent glycine decarboxylase), H protein (a lipoic acid-containing protein), T protein (a tetrahydrofolate-requiring enzyme), and L protein (a lipoamide dehydrogenase). The protein encoded by this gene is the H protein, which transfers the methylamine group of glycine from the P protein to the T protein. Defects in this gene are a cause of nonketotic hyperglycinemia (NKH). Two transcript variants, one protein-coding and the other probably not protein-coding,have been found for this gene. Also, several transcribed and non-transcribed pseudogenes of this gene exist throughout the genome. |
| DLST* | This gene encodes a mitochondrial protein that belongs to the 2-oxoacid dehydrogenase family. This protein is one of the three components (the E2 component) of the 2-oxoglutarate dehydrogenase complex that catalyzes the overall conversion of 2-oxoglutarate to succinyl-CoA and CO(2). Alternatively spliced transcript variants have been found for this gene. |

* Annotation derived from Entrez gene summary (https://www.ncbi.nlm.nih.gov/gene/)

Reference

1. Zhang Z, Ma Y, Guo X, Du Y, Zhu Q, Wang X, et al. FDX1 can Impact the Prognosis and Mediate the Metabolism of Lung Adenocarcinoma. Front Pharmacol. 2021;12:749134.

2. Tsvetkov P, Detappe A, Cai K, Keys HR, Brune Z, Ying W, et al. Mitochondrial metabolism promotes adaptation to proteotoxic stress. Nat Chem Biol. 2019;15(7):681-9.

3. Solmonson A, Faubert B, Gu W, Rao A, Cowdin MA, Menendez-Montes I, et al. Compartmentalized metabolism supports midgestation mammalian development. Nature. 2022;604(7905):349-53.

4. Ji L, Zhao G, Zhang P, Huo W, Dong P, Watari H, et al. Knockout of MTF1 Inhibits the Epithelial to Mesenchymal Transition in Ovarian Cancer Cells. J Cancer. 2018;9(24):4578-85.

5. Masisi BK, El Ansari R, Alfarsi L, Rakha EA, Green AR, Craze ML. The role of glutaminase in cancer. Histopathology. 2020;76(4):498-508.

6. Luo J-P, Wang J, Huang J-H. CDKN2A is a prognostic biomarker and correlated with immune infiltrates in hepatocellular carcinoma. Bioscience reports. 2021;41(10).
