# Supplementary figures and images for: Cuproptosis-related lncRNAs predict the clinical outcome and immune characteristics of hepatocellular carcinoma
Source: Front Genet. 2022 Sep 23;13:972212. doi: 10.3389/fgene.2022.972212 (PMC9538148; doi:10.3389/fgene.2022.972212)

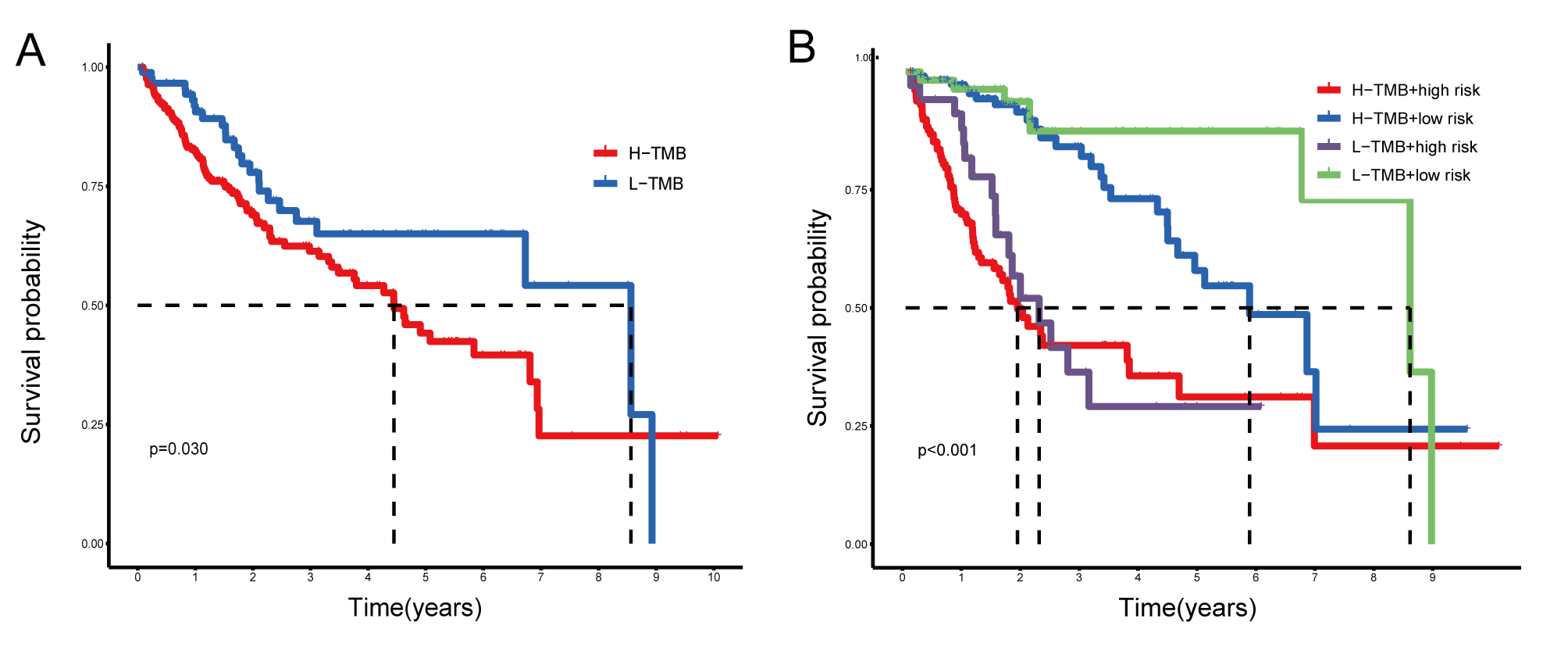

Supplement: Supplementary file 4 [file Image3.TIF]

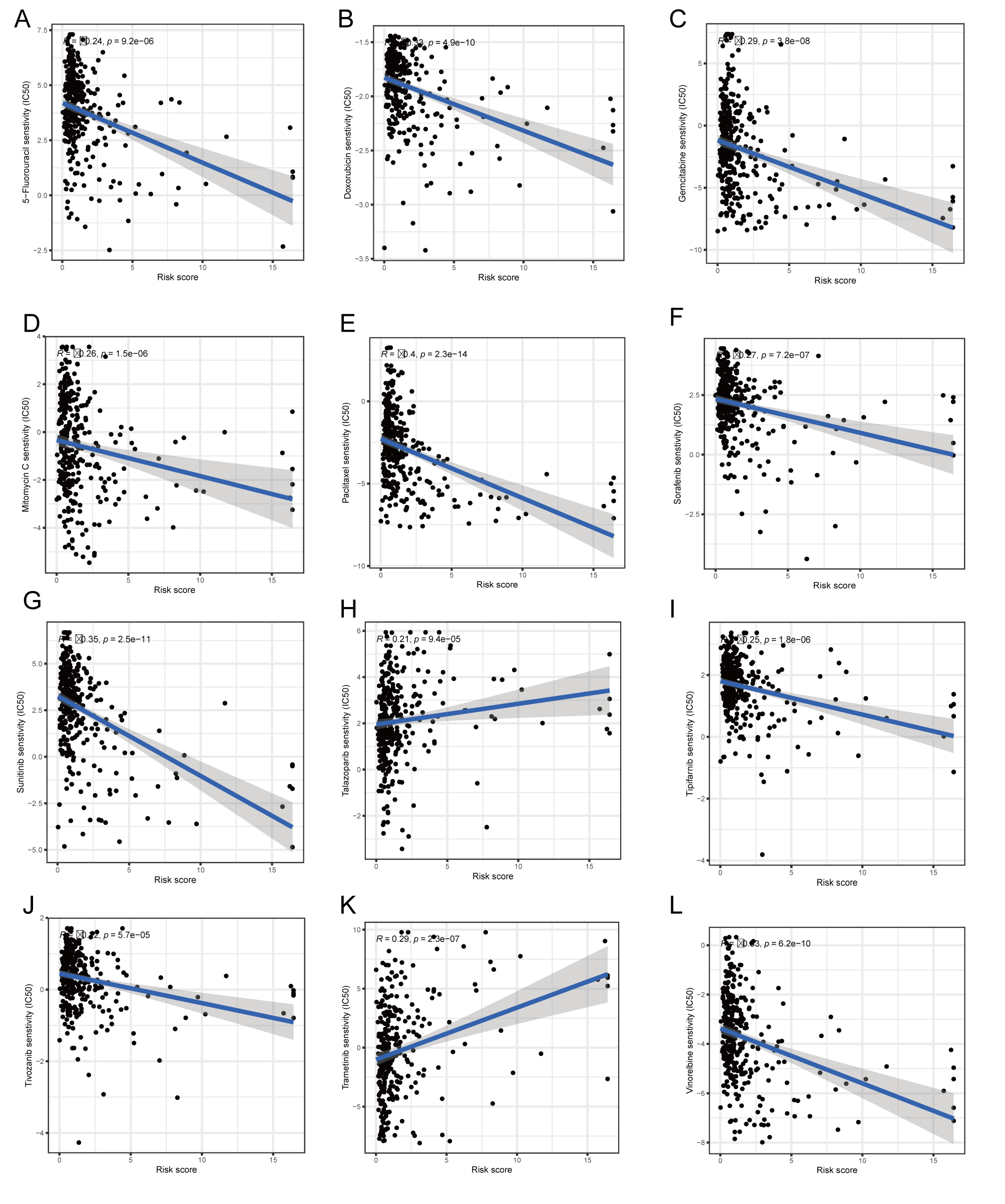

Supplement: Supplementary file 5 [file Image4.TIF]

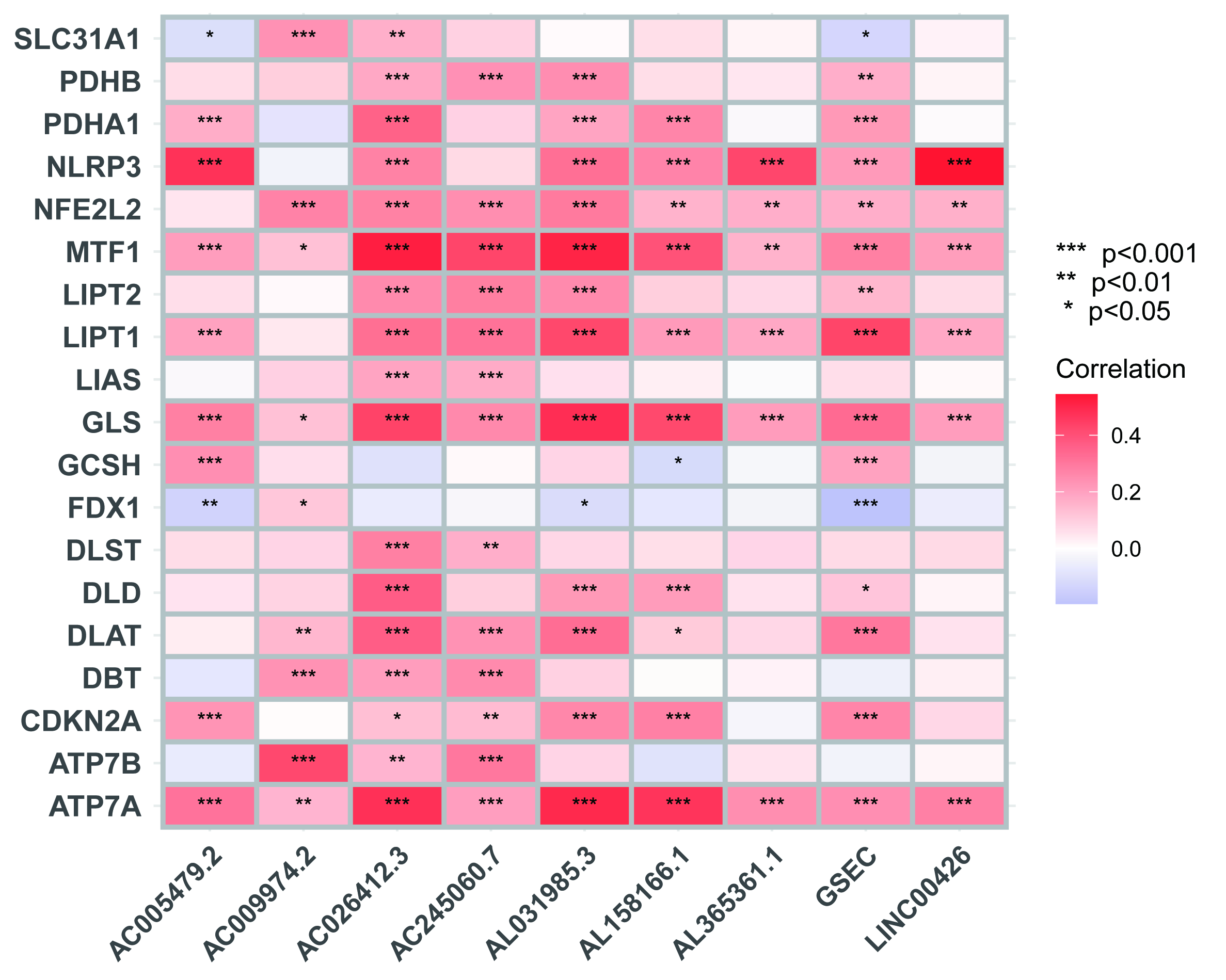

Supplement: Supplementary file 6 [file Image2.TIF]

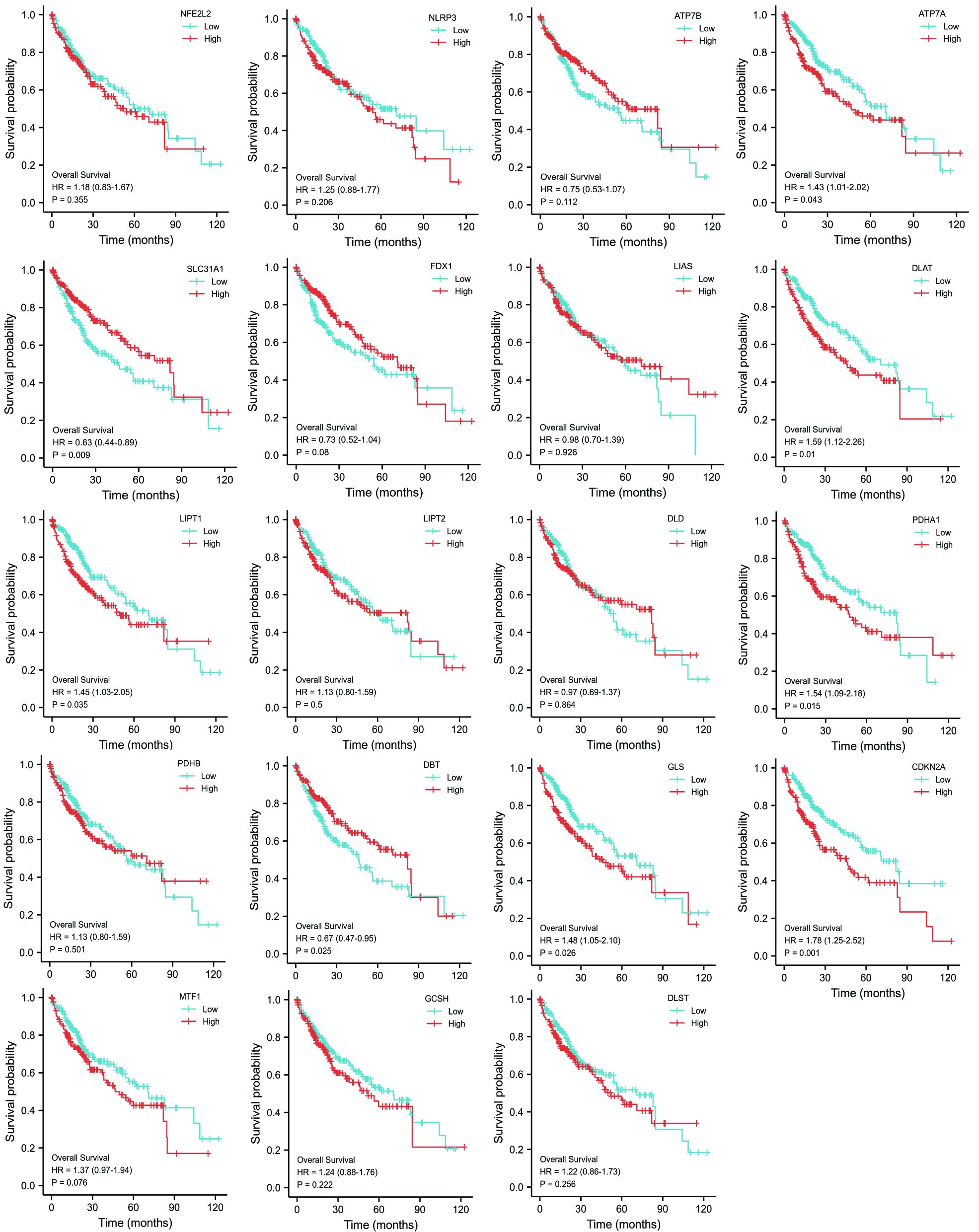

Supplement: Supplementary file 7 [file Image1.TIF]
