# Supplementary material for: Cuproptosis-related lncRNAs predict the clinical outcome and immune characteristics of hepatocellular carcinoma
Source: Front Genet. 2022 Sep 23;13:972212. doi: 10.3389/fgene.2022.972212 (PMC9538148; doi:10.3389/fgene.2022.972212)
Supplement: Supplementary file 9 [file Table5.docx]

Table S5. Clinical characteristics between the low- and high- groups

| Covariates | Type | Total | Test | Train | P value |
| --- | --- | --- | --- | --- | --- |
| age | <=65 | 216(62.97%) | 70(68.63%) | 146(60.58%) | 0.1976 |
|  | >65 | 127(37.03%) | 32(31.37%) | 95(39.42%) |  |
| gender | FEMALE | 110(32.07%) | 29(28.43%) | 81(33.61%) | 0.4164 |
|  | MALE | 233(67.93%) | 73(71.57%) | 160(66.39%) |  |
| grade | G1 | 53(15.45%) | 11(10.78%) | 42(17.43%) | 0.3142 |
|  | G2 | 161(46.94%) | 46(45.1%) | 115(47.72%) |  |
|  | G3 | 112(32.65%) | 37(36.27%) | 75(31.12%) |  |
|  | G4 | 12(3.5%) | 5(4.9%) | 7(2.9%) |  |
| stage | Stage I | 161(46.94%) | 53(51.96%) | 108(44.81%) | 0.0939 |
|  | Stage II | 77(22.45%) | 23(22.55%) | 54(22.41%) |  |
|  | Stage III | 80(23.32%) | 16(15.69%) | 64(26.56%) |  |
|  | Stage IV | 3(0.87%) | 2(1.96%) | 1(0.41%) |  |
| T | T1 | 168(48.98%) | 55(53.92%) | 113(46.89%) | 0.2931 |
|  | T2 | 82(23.91%) | 24(23.53%) | 58(24.07%) |  |
|  | T3 | 76(22.16%) | 16(15.69%) | 60(24.9%) |  |
|  | T4 | 14(4.08%) | 5(4.9%) | 9(3.73%) |  |
| M | M0 | 245(71.43%) | 73(71.57%) | 172(71.37%) | 0.4535 |
|  | M1 | 3(0.87%) | 2(1.96%) | 1(0.41%) |  |
| N | N0 | 239(69.68%) | 71(69.61%) | 168(69.71%) | 1 |
|  | N1 | 3(0.87%) | 1(0.98%) | 2(0.83%) |  |
