# Supplementary material for: Cuproptosis-related lncRNAs predict the clinical outcome and immune characteristics of hepatocellular carcinoma
Source: Front Genet. 2022 Sep 23;13:972212. doi: 10.3389/fgene.2022.972212 (PMC9538148; doi:10.3389/fgene.2022.972212)
Supplement: Supplementary file 10 [file Table4.docx]

**Table S4** Univariate cox results of cuproptosis-related lncRNAs in LIHC.

| id | HR | HR.95L | HR.95H | pvalue |
| --- | --- | --- | --- | --- |
| POLH-AS1 | 1.692645 | 1.093803 | 2.619345 | 0.018155 |
| AC007405.3 | 1.522922 | 1.123321 | 2.064675 | 0.006751 |
| TMCC1-AS1 | 2.785863 | 1.796783 | 4.319403 | 4.68E-06 |
| AC120114.1 | 1.66898 | 1.058227 | 2.63223 | 0.027565 |
| AL355388.1 | 1.85414 | 1.097112 | 3.133531 | 0.021103 |
| AL355574.1 | 1.927035 | 1.350299 | 2.750105 | 0.0003 |
| AC012645.4 | 3.921988 | 1.375561 | 11.18234 | 0.010575 |
| AC098484.4 | 1.944127 | 1.130169 | 3.344306 | 0.016301 |
| AC245060.6 | 2.58478 | 1.129595 | 5.914587 | 0.024545 |
| U47924.3 | 1.828703 | 1.172507 | 2.852141 | 0.007774 |
| AC018529.2 | 2.982321 | 1.151946 | 7.721052 | 0.024359 |
| AC016705.2 | 1.633345 | 1.099138 | 2.427191 | 0.015195 |
| GSEC | 1.971709 | 1.416904 | 2.743755 | 5.65E-05 |
| LINC02038 | 1.499392 | 1.085599 | 2.07091 | 0.013954 |
| PRR7-AS1 | 2.723864 | 1.731652 | 4.2846 | 1.45E-05 |
| AL158166.1 | 1.981616 | 1.484616 | 2.644996 | 3.45E-06 |
| FAM182B | 1.991828 | 1.103326 | 3.595836 | 0.022242 |
| AC004466.2 | 3.217914 | 1.625826 | 6.369052 | 0.000793 |
| ARAP1-AS2 | 3.707593 | 1.114767 | 12.33105 | 0.032585 |
| SAP30-DT | 4.733032 | 1.637068 | 13.68397 | 0.004106 |
| SNHG12 | 1.487797 | 1.134867 | 1.950485 | 0.004031 |
| Z69733.1 | 4.152631 | 1.410369 | 12.22683 | 0.009765 |
| NRAV | 1.908421 | 1.421152 | 2.562758 | 1.73E-05 |
| ATP1A1-AS1 | 1.932796 | 1.165476 | 3.2053 | 0.010671 |
| AL031670.1 | 1.935474 | 1.232258 | 3.039994 | 0.004149 |
| AC004923.4 | 1.796783 | 1.134396 | 2.845946 | 0.012512 |
| BX842570.1 | 1.702976 | 1.172629 | 2.473184 | 0.005167 |
| MCM3AP-AS1 | 3.025116 | 1.434206 | 6.380763 | 0.00365 |
| AC016747.1 | 1.454531 | 1.046658 | 2.021349 | 0.025644 |
| AP000487.1 | 2.809083 | 1.422643 | 5.546681 | 0.002925 |
| AC253576.2 | 1.732587 | 1.117463 | 2.686317 | 0.014037 |
| Z99943.1 | 3.168453 | 1.633486 | 6.145812 | 0.000646 |
| ECI2-DT | 4.385628 | 1.404701 | 13.69241 | 0.010928 |
| AL499602.1 | 1.316144 | 1.132111 | 1.530093 | 0.000351 |
| AC011462.5 | 1.94135 | 1.080081 | 3.489404 | 0.026591 |
| AL606489.1 | 1.286143 | 1.028123 | 1.608917 | 0.027614 |
| AC019080.1 | 5.005146 | 1.801215 | 13.9081 | 0.002012 |
| AC002116.2 | 1.612347 | 1.034648 | 2.512607 | 0.03482 |
| DDX11-AS1 | 2.355772 | 1.284405 | 4.320806 | 0.005628 |
| AL358472.4 | 1.432844 | 1.02621 | 2.000606 | 0.034696 |
| AC087392.3 | 3.870588 | 1.06497 | 14.0675 | 0.039824 |
| AC005479.2 | 2.182201 | 1.514702 | 3.143852 | 2.80E-05 |
| LINC00654 | 1.584929 | 1.037661 | 2.420829 | 0.033087 |
| AC100861.1 | 1.860992 | 1.209413 | 2.863613 | 0.004733 |
| AL513320.1 | 1.590547 | 1.096556 | 2.307077 | 0.014456 |
| LINC00630 | 3.188173 | 1.006812 | 10.09567 | 0.048666 |
| AC114956.2 | 2.926796 | 1.173318 | 7.300777 | 0.021297 |
| AP003390.1 | 1.634869 | 1.151939 | 2.320258 | 0.005927 |
| TRAPPC12-AS1 | 1.404314 | 1.019075 | 1.935185 | 0.037944 |
| SNHG4 | 1.51013 | 1.185495 | 1.923662 | 0.000844 |
| TBC1D8-AS1 | 1.806369 | 1.116863 | 2.921548 | 0.01593 |
| AC125437.1 | 1.70647 | 1.166864 | 2.495611 | 0.005857 |
| AL365361.1 | 0.559902 | 0.33964 | 0.923008 | 0.02296 |
| AC026412.3 | 3.251851 | 1.883177 | 5.615264 | 2.33E-05 |
| FOXD2-AS1 | 1.48348 | 1.195904 | 1.840209 | 0.000334 |
| Z97989.1 | 1.912564 | 1.039144 | 3.520112 | 0.037222 |
| AL031985.3 | 2.162681 | 1.545997 | 3.025355 | 6.68E-06 |
| LINC01775 | 2.095204 | 1.232919 | 3.560559 | 0.006259 |
| MAPKAPK5-AS1 | 1.835962 | 1.312757 | 2.567693 | 0.000385 |
| KDM4A-AS1 | 2.394852 | 1.383481 | 4.145569 | 0.001812 |
| LINC00892 | 0.472074 | 0.245565 | 0.907516 | 0.024387 |
| AL133410.1 | 2.348769 | 1.165826 | 4.732024 | 0.016882 |
| MKLN1-AS | 2.841046 | 1.837003 | 4.393864 | 2.69E-06 |
| GORAB-AS1 | 3.498067 | 1.045434 | 11.70468 | 0.042147 |
| SRD5A3-AS1 | 4.579741 | 1.110868 | 18.88075 | 0.035253 |
| AP001626.1 | 1.711274 | 1.039265 | 2.817815 | 0.034745 |
| LINC00997 | 1.525714 | 1.050664 | 2.215553 | 0.026444 |
| MIR4435-2HG | 2.379366 | 1.548953 | 3.654973 | 7.56E-05 |
| AC107214.1 | 2.309738 | 1.08665 | 4.909481 | 0.029558 |
| AC073842.2 | 1.603917 | 1.083574 | 2.374136 | 0.018221 |
| AC026356.2 | 2.726807 | 1.253937 | 5.929702 | 0.011377 |
| AC073046.3 | 2.213086 | 1.021649 | 4.793966 | 0.043981 |
| AC068205.2 | 1.832771 | 1.098576 | 3.05764 | 0.020341 |
| ZEB1-AS1 | 1.658603 | 1.19774 | 2.296796 | 0.002317 |
| SCAT2 | 1.367474 | 1.01021 | 1.851087 | 0.042794 |
| AL357079.1 | 2.54141 | 1.604627 | 4.025089 | 7.02E-05 |
| LINC00426 | 0.352786 | 0.152424 | 0.816525 | 0.014959 |
| CYTOR | 1.656111 | 1.231228 | 2.227617 | 0.000852 |
| AC108463.2 | 2.075955 | 1.244231 | 3.463658 | 0.005164 |
| AP002449.1 | 1.774277 | 1.132527 | 2.779676 | 0.012305 |
| MED8-AS1 | 1.858081 | 1.260196 | 2.739626 | 0.001764 |
| AC099850.1 | 1.538499 | 1.027092 | 2.304545 | 0.036651 |
| AL021392.1 | 2.199937 | 1.207357 | 4.008529 | 0.010009 |
| LINC00205 | 1.353802 | 1.001258 | 1.830476 | 0.049053 |
| TTC39A-AS1 | 1.812861 | 1.124459 | 2.922708 | 0.014633 |
| AL358394.1 | 1.411624 | 1.089206 | 1.829482 | 0.009164 |
| AL122035.1 | 1.999246 | 1.272751 | 3.14043 | 0.002641 |
| DGUOK-AS1 | 1.606938 | 1.025663 | 2.51764 | 0.038398 |
| NRSN2-AS1 | 1.584996 | 1.058177 | 2.374094 | 0.025465 |
| AL354824.2 | 3.442825 | 1.687457 | 7.024205 | 0.000679 |
| AL357079.3 | 1.803189 | 1.244056 | 2.613621 | 0.001852 |
| AC048344.4 | 2.489521 | 1.352746 | 4.581579 | 0.003381 |
| AC010973.2 | 2.07753 | 1.193855 | 3.615287 | 0.009686 |
| PICSAR | 1.33614 | 1.046267 | 1.706323 | 0.020209 |
| SNHG3 | 1.400853 | 1.140721 | 1.720306 | 0.001299 |
| AC124016.1 | 1.666917 | 1.036453 | 2.680885 | 0.035061 |
| AL121748.1 | 3.777487 | 1.314379 | 10.85639 | 0.013607 |
| FAM225A | 2.573948 | 1.240465 | 5.340906 | 0.011131 |
| LINC01772 | 3.112558 | 1.381001 | 7.015214 | 0.006171 |
| AC027097.1 | 1.981471 | 1.250216 | 3.140439 | 0.00361 |
| AC011815.1 | 1.584544 | 1.010881 | 2.483756 | 0.044733 |
| AC104024.4 | 2.799757 | 1.045298 | 7.498951 | 0.040551 |
| AC092667.1 | 1.753097 | 1.069457 | 2.873747 | 0.025997 |
| AL683813.1 | 2.035089 | 1.163491 | 3.559623 | 0.012746 |
| RUFY1-AS1 | 2.93046 | 1.122863 | 7.647945 | 0.02804 |
| AC009133.1 | 2.247921 | 1.295898 | 3.899343 | 0.003948 |
| SBF2-AS1 | 1.595532 | 1.118118 | 2.276792 | 0.010012 |
| RHOQ-AS1 | 2.96997 | 1.195523 | 7.378127 | 0.019047 |
| AL138756.1 | 1.440082 | 1.016809 | 2.039555 | 0.039992 |
| SYNGAP1-AS1 | 3.129475 | 1.207631 | 8.109773 | 0.01886 |
| ARNILA | 3.393609 | 1.508457 | 7.634673 | 0.00314 |
| AC137932.3 | 2.308086 | 1.19216 | 4.468578 | 0.013086 |
| AC009961.1 | 2.346871 | 1.252292 | 4.398176 | 0.007768 |
| AL049840.4 | 1.815849 | 1.042699 | 3.162281 | 0.035058 |
| C2orf27A | 2.287116 | 1.555061 | 3.363791 | 2.63E-05 |
| STX18-AS1 | 3.432412 | 1.130261 | 10.42366 | 0.029554 |
| AC006252.1 | 1.827716 | 1.164149 | 2.869517 | 0.008783 |
| WAC-AS1 | 1.4313 | 1.042763 | 1.964608 | 0.02648 |
| SPRY4-AS1 | 3.028958 | 1.820878 | 5.038551 | 1.97E-05 |
| SNHG26 | 1.922883 | 1.330478 | 2.779062 | 0.000502 |
| PLBD1-AS1 | 1.627564 | 1.167914 | 2.268116 | 0.004019 |
| AP001029.1 | 1.736902 | 1.179825 | 2.557012 | 0.005141 |
| AP001469.3 | 1.575291 | 1.117213 | 2.221191 | 0.009537 |
| LINC01094 | 1.881039 | 1.222285 | 2.894831 | 0.004072 |
| AL365436.2 | 3.066251 | 1.111497 | 8.458771 | 0.030454 |
| AL109627.1 | 1.609828 | 1.160592 | 2.232954 | 0.004343 |
| SNHG20 | 1.575274 | 1.082796 | 2.291741 | 0.017508 |
| AL359878.1 | 4.748345 | 1.919871 | 11.7439 | 0.000747 |
| AC087294.1 | 2.84203 | 1.216689 | 6.638616 | 0.015818 |
| AL117335.1 | 1.598441 | 1.203473 | 2.123033 | 0.0012 |
| LINC00628 | 1.72974 | 1.028378 | 2.909437 | 0.038882 |
| LYRM4-AS1 | 1.526848 | 1.02008 | 2.285374 | 0.039727 |
| SAP30L-AS1 | 7.01548 | 1.812188 | 27.15886 | 0.00479 |
| AL590705.3 | 1.638698 | 1.183621 | 2.268742 | 0.002924 |
| AC106786.1 | 1.539174 | 1.007318 | 2.351847 | 0.046188 |
| LINC02298 | 1.537896 | 1.059985 | 2.231281 | 0.023405 |
| LINC00862 | 1.728033 | 1.186989 | 2.51569 | 0.00431 |
| OSMR-AS1 | 2.533892 | 1.044909 | 6.144655 | 0.039671 |
| AL445231.1 | 3.508271 | 1.121151 | 10.97797 | 0.031049 |
| AL353572.4 | 1.269919 | 1.094525 | 1.473418 | 0.001627 |
| ALMS1-IT1 | 1.583893 | 1.014493 | 2.472877 | 0.043046 |
| AC012443.2 | 5.454647 | 2.320308 | 12.82294 | 0.0001 |
| AP001453.2 | 2.782692 | 1.412007 | 5.48395 | 0.003109 |
| AL451069.3 | 1.502142 | 1.138527 | 1.981887 | 0.00401 |
| AC022211.3 | 1.964744 | 1.175625 | 3.283545 | 0.009953 |
| LINC01184 | 1.577096 | 1.085664 | 2.290978 | 0.016785 |
| AC108463.3 | 2.942606 | 1.386631 | 6.244584 | 0.004932 |
| Z92544.1 | 3.079666 | 1.500918 | 6.319031 | 0.00216 |
| AC012313.8 | 3.642416 | 1.119487 | 11.85114 | 0.031755 |
| LINC01011 | 1.75595 | 1.199928 | 2.569622 | 0.003753 |
| HMGN3-AS1 | 1.723499 | 1.146709 | 2.590412 | 0.008833 |
| AC040970.1 | 1.286181 | 1.049076 | 1.576876 | 0.015487 |
| AL024498.1 | 2.215891 | 1.040886 | 4.717302 | 0.039026 |
| ZNF337-AS1 | 1.724123 | 1.023521 | 2.90429 | 0.040624 |
| AC007996.1 | 1.419652 | 1.097769 | 1.835915 | 0.007563 |
| AC009974.2 | 0.246592 | 0.064047 | 0.949415 | 0.041807 |
| AC079174.2 | 1.637466 | 1.038586 | 2.58168 | 0.033758 |
| AC073611.1 | 5.737234 | 1.224149 | 26.88876 | 0.026652 |
| PVT1 | 1.852098 | 1.165381 | 2.943472 | 0.009122 |
| AC016394.2 | 1.346438 | 1.030582 | 1.759099 | 0.029197 |
| AL355488.1 | 1.403752 | 1.084629 | 1.816766 | 0.009957 |
| THUMPD3-AS1 | 1.503238 | 1.022691 | 2.209588 | 0.038067 |
| LINC01711 | 1.814816 | 1.105562 | 2.979077 | 0.018433 |
| AC137561.1 | 1.924607 | 1.194405 | 3.10122 | 0.00715 |
| AC026356.1 | 2.168799 | 1.447767 | 3.248925 | 0.000174 |
| AC090018.2 | 3.043913 | 1.200638 | 7.717073 | 0.019017 |
| AL133370.1 | 1.343072 | 1.114506 | 1.618515 | 0.001942 |
| AC025265.3 | 2.285211 | 1.333483 | 3.916202 | 0.002637 |
| AC024560.4 | 1.668152 | 1.009819 | 2.755673 | 0.045704 |
| AC022167.2 | 2.112568 | 1.057656 | 4.219653 | 0.03411 |
| RNF216P1 | 1.832946 | 1.252045 | 2.683363 | 0.001834 |
| AC087289.5 | 2.106155 | 1.111036 | 3.99257 | 0.022452 |
| AL133477.1 | 1.566222 | 1.064191 | 2.305087 | 0.022876 |
| MELTF-AS1 | 1.276234 | 1.004685 | 1.621179 | 0.045689 |
| KTN1-AS1 | 2.040366 | 1.020203 | 4.080651 | 0.043745 |
| AL078644.1 | 1.786825 | 1.108623 | 2.879918 | 0.017154 |
| AC026401.3 | 1.392448 | 1.121324 | 1.729127 | 0.002732 |
| AC025442.2 | 3.182319 | 1.168363 | 8.667811 | 0.023554 |
| LINC02449 | 3.066043 | 1.473292 | 6.38069 | 0.002733 |
| TRAF3IP2-AS1 | 2.772322 | 1.372217 | 5.600988 | 0.004485 |
| AC123768.1 | 3.053939 | 1.179765 | 7.90542 | 0.021413 |
| AC021851.1 | 1.97033 | 1.207651 | 3.214674 | 0.00662 |
| AL513327.1 | 2.037645 | 1.197082 | 3.468431 | 0.008721 |
| AC083855.2 | 1.385998 | 1.086147 | 1.768627 | 0.008682 |
| AF241728.2 | 3.044259 | 1.027023 | 9.023666 | 0.044637 |
| LINC02870 | 1.508858 | 1.197211 | 1.901629 | 0.000493 |
| AC022211.1 | 3.841272 | 1.386475 | 10.64236 | 0.009641 |
| AL603839.2 | 1.682306 | 1.119226 | 2.52867 | 0.01236 |
| AC109449.1 | 4.233424 | 1.672414 | 10.71617 | 0.002325 |
| AC022007.1 | 1.558692 | 1.065516 | 2.280135 | 0.022199 |
| AC018645.3 | 1.562137 | 1.134576 | 2.150823 | 0.006261 |
| AC107959.3 | 1.449756 | 1.168063 | 1.799383 | 0.000754 |
| AC138028.6 | 2.184815 | 1.244899 | 3.834381 | 0.006464 |
| AC007285.1 | 3.151313 | 1.259744 | 7.883169 | 0.014145 |
| AL137127.1 | 2.458575 | 1.10594 | 5.465568 | 0.027314 |
| AL161729.4 | 1.586159 | 1.050248 | 2.395532 | 0.028306 |
| SNHG1 | 1.387856 | 1.089343 | 1.768171 | 0.00799 |
| UBE2D3-AS1 | 1.558595 | 1.026205 | 2.367186 | 0.037408 |
| AL117336.2 | 1.681235 | 1.218797 | 2.319133 | 0.001548 |
| LINC01106 | 1.883833 | 1.190618 | 2.98066 | 0.006826 |
| AC245060.7 | 3.90089 | 1.896683 | 8.022923 | 0.000216 |
| WARS2-AS1 | 1.63272 | 1.064156 | 2.505061 | 0.024789 |
| AL442125.1 | 4.011367 | 1.818543 | 8.848327 | 0.000578 |
| AP000640.1 | 4.565061 | 1.675209 | 12.4401 | 0.002991 |
| AC116351.1 | 1.277736 | 1.066853 | 1.530303 | 0.007741 |
| SCTR-AS1 | 1.203044 | 1.01627 | 1.424145 | 0.031758 |
| ARRDC1-AS1 | 1.483572 | 1.009602 | 2.180055 | 0.044577 |
| AC100778.2 | 1.907682 | 1.148953 | 3.167452 | 0.012536 |
| AC006026.3 | 1.298961 | 1.026636 | 1.643524 | 0.029335 |
| LINC02709 | 1.720713 | 1.225926 | 2.415197 | 0.001704 |
| Z97832.2 | 1.980918 | 1.127967 | 3.478857 | 0.017357 |
